# Relationship between Multimorbidity and Disability in Elderly Patients with Coexisting Frailty Syndrome

**DOI:** 10.3390/ijerph19063461

**Published:** 2022-03-15

**Authors:** Maria Jędrzejczyk, Weronika Foryś, Michał Czapla, Izabella Uchmanowicz

**Affiliations:** 1 Department of Nursing and Obstetrics, Faculty of Health Sciences, Wroclaw Medical University, 51-618 Wrocław, Poland; maria.jedrzejczyk@umw.edu.pl (M.J.); izabella.uchmanowicz@umw.edu.pl (I.U.); 2 Harvard Division of Continuing Education, Harvard University, Cambridge, MA 02138-3722, USA; wef651@g.harvard.edu; 3 Laboratory for Experimental Medicine and Innovative Technologies, Department of Emergency Medical Service, Wroclaw Medical University, 51-616 Wroclaw, Poland; 4 Institute of Heart Diseases, University Hospital, 50-566 Wroclaw, Poland; 5 Faculty of Nursing, Group in Research in Care (GRUPAC), University of La Rioja, 26006 Logroño, Spain

**Keywords:** multimorbidity, functional disability, old age, frailty syndrome

## Abstract

Background: Multimorbidity is a common problem worldwide. It carries the risk of reduced quality of life, disability, frequent hospitalizations, and death. The present study was designed to assess the relationships that exist between multimorbidity and disability in elderly patients. Methods: The study included 100 patients and was conducted between October 2020 and January 2021. Inclusion criteria included age >65 years, presence of a minimum of two comorbidities in the subject, and consent to participate in the study. Standardized survey instruments such as Tilburg Frailty Indicator (TFI), Charlson Comorbidities Index (CCI), Assessment of Basic Activities of Daily Living—Katz Scale (ADL), and Assessment of Complex Activities of Daily Living—Lawton Scale (IADL) were used in the study. Results: The majority of the subjects (92) had a frailty syndrome (TFI). A small group of respondents (8%) suffered from severe comorbidities (CCI). Among the subjects surveyed, 71% maintained full function in performing simple activities of daily living (ADL), while 29% demonstrated moderate disability on the scale. Full independence in performing complex activities of daily living (IADL) was present in 33% of the respondents, and 67% were partially independent. Independence in complex activities of daily living (IADL) was significantly higher in patients with fewer comorbidities. The severity of comorbidities (CCI) had a significant effect on the decrease in the level of independence (ADL and IADL). Independence in performing complex activities (IADL) was worse among older patients. Conclusions: An increase in the number of comorbidities contributes to a decrease in the level of performance of complex activities of daily living. The severity of comorbidities significantly reduces the level of independence of the subjects in simple and complex activities of daily living. In patients with a higher level of independence in performing simple and complex activities, the co-occurrence of frailty syndrome was less severe. As the age of the subjects increases, the frequency in which they show moderate dependence on third parties in performing complex activities of daily living increases.

## 1. Introduction

The aging process has been a big challenge in geriatric care. The aging process causes the human body to change irreversibly. Changes caused by aging are correlated with the increased risk of the onset of chronic diseases and the limitation of functional efficiency [1,2]. Older people are also the fastest and most significantly growing social group [3].

Multi-disease is the co-existence of two or more chronic diseases in one person. This phenomenon increases with age and is associated with high mortality, decreased quality of life, and limited of the functional state [4,5]. People with multiple chronic conditions have a higher risk of hospitalization and their need for medical care increases [6,7]. Socio-demographic factors predominantly influence the occurrence of various diseases. Older people living in the countryside and people with low income often have worse access to medical care and knowledge [7].

Functional disability is a state of partial or complete dependence and dependence on the performance of life activities. This phenomenon often occurs in the elderly. It appears to result from the aging organism and the coexistence of many diseases [8,9]. Disability risk factors include gender, age, socioeconomic status, and chronic diseases. Each case should be considered individually [10]. The risk of disability increases with age, and the most significant functional limitations are observed in people aged 80 and over; it results from limiting the operating organ reserves [11,12].

Frailty syndrome, also known as the weakness syndrome, is a geriatric condition that reduces the body’s reserves. Weakness significantly accelerates the deterioration of the fitness of the elderly. Therefore, people suffering from this syndrome are at risk of developing adverse health effects, such as falls, frequent hospitalizations, disability and dependence on other people, and in the worst case, death [13,14,15].

The purpose of this study was to find the associations that exist between the variables described above—elderly age, multimorbidity, disability, and frailty syndrome.

## 2. Materials and Methods

### 2.1. Participants and Settings

The research was carried out among patients hospitalized at the University Teaching Hospital in Wrocław, in the Department of Cardiology, due to cardiovascular diseases (hypertension, coronary artery disease, arrhythmias, heart failure). The study was conducted between October 2020 and January 2021. Participation in the study was voluntary and anonymous. Researchers preliminarily chose 176 people to participate in the study based on the inclusion criteria. The inclusion criteria for the study were: elderly age—65 years or older; and the presence of multimorbidity—patients diagnosed with two or more diseases. Out of 176 patients meeting the inclusion criteria, 76 failed to fill out the questionnaires correctly and were excluded from the study. The final sample consisted of 100 subjects (48 men, 52 women) with a mean age (71.89 ± 5.46).

### 2.2. Research Tools

The study’s authors collected clinical data using the patient’s medical database. The following standardized questionnaires were also used: (1) Tilburg Frailty Indicator (TFI), (2) Charlson Coexistence Index (Charlson Comorbidity Index—CCI), (3) Assessment Scale for Basic Life Activities—Katz Scale (ADL), (4) Assessment Scale for Complex Daily Life Activities—Lawton Scale (IADL).

#### 2.2.1. Tilburg Frailty Indicator (TFI)

The Tilburg Frailty Indicator (TFI) tool can be used to assess frailty syndrome by taking into account the physical, psychological, and social domains. The questionnaire consists of two parts—part A contains 10 questions that concern socio-demographic characteristics (including gender, age, marital status, country of birth, education, monthly income, etc.) and part B considers the attributes of frailty syndrome. There are 15 questions in part B—8 questions from the physical domain, four questions from the mental field, three questions from the social domain. The total score is between 0 and 15 points. Frailty syndrome is diagnosed when TFI is five or above. The higher the overall score obtained, the higher the weakness index [16,17].

#### 2.2.2. Charlson’s Comorbidity Index (CCI)

The Charlson Coexistence Index (CCI) is a scale that helps to assess a patient’s burden of comorbidities. It allows calculation of the risk of death within one year based on comorbidities and the current health condition. The scale includes 19 conditions. Each of them was assigned a severity level from 1 to 6 points. Based on the sum of the CCI scores, the burden of comorbidities can be divided into three grades: mild—1–2 points, moderate—3–4 points, severe—≥ 5 points; it also means a very high risk of death within one year [18]. When adding an age criterion to the scale, the age–comorbidity score will help determine the survival percentage for the next ten years. For each decade over 50, 1 point is awarded. The maximum number of points is 4 for age equal to or greater than 80 [19,20].

#### 2.2.3. Activity of Daily Living (ADL)

The ADL scale is commonly used in the comprehensive geriatric assessment to assess performance. It contains six questions concerning patients’ independence in carrying out basic life activities, such as washing, getting dressed, using the toilet, moving around, controlling the sphincter, and eating meals. The interpretation of the results is as follows: 0–2 points—significant disability; 3–4 points—moderate disability; 5–6 points—full efficiency. A maximum of 6 points can be obtained [21].

#### 2.2.4. Instrumental Activities of Daily Living (IADL)

The IADL is a tool for assessing a patient's ability to exercise complex activities of daily living. It contains eight questions, which cover the span of phone usage, independent travel, shopping, preparing meals, doing housework, laundry, making repairs, self-medicating, and managing money. The IADL score is in the range of 8–24 points, and higher the results indicate greater patient independence. The IADL scoring system does not include point-range criteria indicating high and low patient independence. However, it is often assumed that the maximum result (24 points) means complete independence and the minimum results (8 points) total dependence. Results in-between the maximum and the minimum results indicate partial independence [22].

### 2.3. Data Analyses

A significance level of 0.05 was adopted in the analysis. All *p* values < 0.05 were interpreted as showing significant relationships. Comparison of the values of quantitative variables in two groups was performed using the Mann–Whitney test. Comparison of the values of quantitative variables in three and more groups was performed using the Kruskal–Wallis test. After detecting statistically significant differences, post hoc analysis with Dunn’s test was performed to identify statistically significantly different groups. Correlations between quantitative variables were analyzed using the Spearman correlation coefficient. Multivariate analysis of the influence of many variables on the quantitative variable was performed using the linear regression method. The results are presented as the values of the regression model parameters with a 95% confidence interval. One- and multivariate analysis of the influence of many variables on the binary variable was performed using the logistic regression method. The results are presented as OR parameter values with a 95% confidence interval. The analysis was conducted using the R software (R Foundation for Statistical Computing, Vienna, Austria), version 4.0.4. [23].

## 3. Results

### 3.1. Characteristics of the Studied Group

One hundred patients with multiple diseases participated in the study. The respondents were 48% men and 52% women. The average age of the respondents was 72 years. The most numerous group were people living with a spouse or partner—48%; single people constituted 19%. Every second person earned more than PLN 2100 per month, 30% of respondents earned between 1800 and 2100, and only 4% earned the lowest amount—between PLN 900 and 1200. We obtained the number of comorbidities using clinical data. Of the subjects tested, 20% had two diseases, 59% had three chronic diseases, and 21% had four or more diseases. Brittleness syndrome occurred in 92% of respondents. More than half of the respondents had mild comorbidities on the CCI scale—64%; only 8% suffered from severe comorbidities. Seventy-one percent of the respondents remained fully functional, and 29% were moderately disabled on the ADL scale. Thirty-three percent of respondents had complete independence on the IADL scale, and 67% were partially independent. The socio-demographic and clinical data and the results of standardized tools are presented in Table 1.

### 3.2. The Impact of Individual Variables on the Tilburg Frailty Indicator (TFI)

Due to the prevalence of frailty syndrome in most patients, it is impossible to investigate the effect of comorbidities on the occurrence of frailty syndrome. Therefore, the influence of multiple morbidities (CCI) on the overall result of TFI, physical components, psychological components, and social components was checked. An analysis of the Spearman correlation between the frailty score (TFI) and the Charlson Coexistence Index (CCI), the Katz score (ADL), and the Lawton score (IADL) was performed. No significant correlations were found between TFI and CCI (all *p* > 0.05). The correlation analyses showed that the score on the scale of physical features of brittleness decreased due to the ADL scale increase (greater independence). It was also revealed that the higher the IADL score (greater autonomy), the lower the overall TFI score (lower severity of the brittleness syndrome) and the score on the scale of physical components of brittleness (Table 2).

### 3.3. Relationship between the Number of Comorbidities and the IADL Score

An analysis of the correlation between the number of comorbidities and the IADL score was also performed using the Kruskal-Wallis test and post hoc analysis (Dunn’s test). Independence in complex daily activities was significantly greater in the group with two or three diseases than those with four or more diseases (Table 3).

### 3.4. Relationship between the Severity of Comorbidities

Another analysis performed was the Spearman correlation between the severity of comorbidities (CCI) and the level of independence in performing simple (ADL) and complex everyday activities (IADL). It has been shown that the greater the severity of comorbidities, the lower the independence in simple (ADL) and complex daily activities (IADL) (Table 4).

### 3.5. The Influence of Age on the IADL Score

Then, an analysis of the Spearman correlation between the age of the respondents and the IADL scale result was performed. It has been shown that the older the age, the less independent in complex everyday activities (Table 5).

### 3.6. Results of Univariable and Multivariable Regression Analysis—Multimorbidity and IADL

A one-way and multi-factor analysis of the relationship between multimorbidity and disability in performing complex everyday life activities (IADL) was also carried out. During the univariate analysis, logistic regression models (separate for each of the analyzed features) showed that significant (*p* ˂ 0.05) predictors of the chance of only partial independence are living with family (OR = 9) and remuneration of PLN 1801–2100 (OR = 0.316). Living with a family increases the chances of only partial independence nine times compared to living alone. A salary of PLN 1801–2100 lowers the chances of only partial independence by 68.4% compared to a salary of over PLN 2100.

The multivariate logistic regression model showed that a significant (*p* ˂ 0.05) independent predictor of the chance of only partial independence is living with family (OR = 21.485), so it increases the likelihood of only partial independence 21.485 times compared to living alone (Table 6).

### 3.7. Results of Univariable and Multivariable Regression Analysis—Multimorbidity and ADL

Then, univariate and multivariate analyses of the relationship between multimorbidity and disability in basic life activities (ADL) were performed. The univariate analysis showed that the predictors of the odds of only moderate disability are living with family (OR = 7.083), thus increasing the odds of only mild disability 7.083 times compared to living alone; and remuneration PLN 1801–2100 (OR = 0.3), reducing the chances of only a moderate disability by 70.0% concerning salary of over PLN 2100. The multivariate logistic regression model showed that none of the analyzed features are a significant independent predictor of the odds of only moderate disability (as all *p* > 0.05) (Table 7).

## 4. Discussion

The presence of multiple diseases in elderly patients may significantly reduce efficiency, contributing to the phenomenon of dependence on third parties in performing complex and straightforward everyday activities. In the conducted research, disability in performing simple daily activities occurred in 29% of respondents, and a partial disability in complex activities of everyday life in 67% of respondents. Disability is also influenced by significant geriatric syndromes and the increasingly frequent frailty syndrome [24].

The relationship between polymorphism and frailty syndrome is challenging to prove. No statistically significant associations were found between the frailty syndrome and multimorbidity in the authors’ research. Similarly, studies conducted in the elderly population in Mexico by Rivera-Almaraz et al. did not show any association between weakness and multiple diseases [25]. Additionally, a study by Fried et al. showed that 26.6% of subjects with the frailty syndrome were not diagnosed with multiple diseases. These results indicate an independent development of weakness [26].

Various factors often limit functional performance in simple and complex activities in the elderly. In this study, the relationship between disability and frailty syndrome was demonstrated. Efficiency in daily activities, assessed using the ADL scale, correlates significantly with the physical components of frailty syndrome. In contrast, performance in complex activities, assessed using the IADL scale, correlates with the overall TFI score and frailty syndrome’s physical components. This means that in patients with higher ADL and IADL scores, the severity of the frailty syndrome was less severe. In many other studies, conducted by Rivera-Almaraz et al. [25], Papachristou et al. [27], Avila-Funes et al. [28], similar correlations were found between the frailty syndrome and performance scores on the ADL and IADL scales. In the study conducted by Avila-Funes et al., it was additionally noted that weakness was associated with functional disability and hospitalizations that occurred in the period before the study [28]. On the other hand, the work of Papachristou et al. found that weakness and its determinants may affect the incidence of disability in the elderly and increase the frequency of falls and mortality [27].

The present study showed a significant correlation between the number of chronic diseases and the reduction in functional capacity in complex activities of daily living, assessed using the IADL scale. In patients with an increasing number of multiple diseases, the independence of these activities was decreased. It was significantly higher in people with two or three comorbidities than in the group with four or more diseases. Research showed that the higher the severity of comorbidities, the lower the level of independence in everyday and complex activities. Similar results were obtained in the studies carried out by St John et al. [29], Boeckxstaens et al. [30], and Wang et al. [31]. In the research work of St John et al., it was proved that multimorbidity predicts deterioration of the functional state in the elderly, thus leading to death. The occurrence of disability is a symptom of deteriorating health due to the worsening of multiple diseases. Doctors should consider that comorbidities carry a risk of disability over time. In addition, a study by St John et al. indicated that comprehensive geriatric care reduces the risk of functional deterioration, frequent hospitalizations, and the use of nursing homes [29]. A study by Wang et al. suggested that many diseases may be related to disability. Specific patterns have also been noted in cardiometabolic and degenerative psychiatric illnesses that are particularly strongly associated with functional disabilities among the elderly. The results obtained in the study provided information that will help to develop prevention of the development of chronic diseases and functional disability [31].

With age, the biological reserves of the organism decrease, which contribute to limitations in human functioning and the risk of disability and death. The authors’ research shows that the aging process of the patients decreased their functional ability to perform complex everyday life activities. Similar results were obtained in the studies of St John et al. [28] and Su P. et al. [32].

This study also showed the influence of some socio-demographic data on the incidence of disability. The high earnings of the respondents had an impact on the occurrence of a higher independence score on the ADL and IADL scales. On the other hand, living with family increased the risk of reduced functionality compared to living alone. A study by Su P. et al. showed that women with low incomes more often experienced disability resulting from the IADL scale. Older people living alone had better fitness than those living with relatives [32].

A more extensive research group should be gathered when planning future research, and most treatment centers should be included. This will allow for a considerable diversification of the statistical analysis results and the testing of new hypotheses. Measuring multiple morbidities will remain a challenge as assessing the scales are too general and complex. Further research on comorbidities and disabilities in elderly patients could bring many benefits in preventing chronic disease and preventing reduced performance in the elderly.

### Study Limitations

This study was performed at one site only. In addition, the research group was not large—as many as 76 patients incorrectly completed the questionnaires, so 100 people were included in the study. In such a small research group, it was not possible to identify several relationships, e.g., between the gender of people with multiple diseases and independence in performing complex activities of everyday life. There was also no knowledge about the duration of comorbidities, which may affect functional limitations and weaknesses.

## 5. Conclusions

The increase in the number of comorbidities contributes to reducing the fitness level in the performance of complex everyday life activities. The severity of comorbidities significantly reduces the level of independence of the respondents in the field of complex and straightforward daily activities. In patients with a higher level of independence in performing simple and complex tasks, the coexistence of frailty syndrome was less pronounced. As the age of the subjects increases, the frequency in which they show moderate dependence on third parties in carrying out complex activities of daily life increases.

## Figures and Tables

**Table 1:** Socio-demographic and clinical characteristics of the study group.

Parameter		Total (N = 100)
Sex	Women	52 (52%)
	Men	48 (48%)
Age	Up to 70 years	49 (49%)
	71–75 years	30 (30%)
	Over 75 years	21 (21%)
Living	Alone	19 (19%)
	With your spouse or partner	48 (48%)
	With family	33 (33%)
Salary	PLN 2101 or more	50 (50%)
	PLN 1801–2100	30 (30%)
	PLN 1501–1800	11 (11%)
	PLN 1201–1500	5 (5%)
	PLN 901–1200	4 (4%)
Chronic diseases	2 diseases	20 (20%)
	3 diseases	59 (59%)
	4 or more diseases	21 (21%)
TFI	No frailty syndrome	8 (8%)
	Frailty syndrome	92 (92%)
CCI	Mild worsening of comorbidities	64 (64%)
	Moderate aggravation of comorbidities	28 (28%)
	Severe aggravation of comorbidities	8 (8%)
ADL	Moderate disability	29 (29%)
	Full efficiency	71 (71%)
IADL	Partial independence	67 (67%)
	Full independence	33 (33%)

PLN—Polish Zloty; 1 PLN = 0.22 EUR; TFI—Tilburg Frailty Indicator; CCI—Charlson’s Comorbidity Index; ADL—Activities of Daily Living; IADL—Instrumental Activities of Daily Living.

**Table 2:** Coefficient of correlation between the frailty score (TFI) and the Charlson Coexistence Index (CCI), the Katz score (ADL) and the Lawton score (IADL).

TFI	CCI	ADL	IADL
TFI’s overall result	r = 0.083. *p* = 0.412	r = −0.166. *p* = 0.099	r = −0.261. *p* = 0.009
Physical ingredients	r = 0.112. *p* = 0.269	r = −0.324. *p* = 0.001	r = −0.573. *p* < 0.001
Psychological components	r = 0.014. *p* = 0.887	r = 0.033. *p* = 0.743	r = 0.102. *p* = 0.312
Social components	r = 0.081. *p* = 0.426	r = 0.002. *p* = 0.986	r = 0.055. *p* = 0.588

TFI—Tilburg Frailty Indicator; CCI—Charlson’s Comorbidity Index; ADL—Activities of Daily Living; IADL—Instrumental Activities of Daily Living.

**Table 3:** Relationship between the number of comorbidities and the IADL score.

IADL [Points]	Comorbidities			*p*-Value
	2 Diseases (N = 20)-A	3 Diseases (N = 59)-B	4 or More Diseases (N = 21)-C	
Mean ± SD	21.35 ± 3.07	20.63 ± 3.49	18.52 ± 3.89	*p* = 0.041
Median	22.5	21	19	
Quartiles	18.75–24	19–24	15–21	A.B > C

IADL—Instrumental Activities of Daily Living.

**Table 4:** Relationship between the severity of comorbidities (CCI) and the ADL and IADL scores.

Tested Features	Spearman’s Correlation Coefficient	*p*-Value
CCI and ADL	−0.403	*p* < 0.001
CCI and IADL	−0.242	*p* = 0.015

CCI—Charlson’s Comorbidity Index; ADL—Activities of Daily Living; IADL—Instrumental Activities of Daily Living.

**Table 5:** Influence of age on the IADL result.

Tested Features	Spearman’s Correlation Coefficient	*p*-Value
Age and IADL	−0.387	*p* < 0.001

IADL—Instrumental Activities of Daily Living.

**Table 6:** Results of univariable and multivariable regression analysis—multimorbidity and IADL.

Characteristic		N (%)	One-Factor Models				Multivariable Model			
			OR	95% CI		*p*-Value	OR	95% CI		*p*-Value
Sex	Women	36 (69.23%)	1	ref.			1	ref.		
	Men	31 (64.58%)	0.81	0.352	1.867	0.622	0.933	0.3	2.899	0.905
Age	Up to 70 years old	27 (55.10%)	1	ref.			1	ref.		
	71–75 years old	19 (63.33%)	1.407	0.554	3.574	0.472	1.019	0.34	3.056	0.973
	Over 75 years old	21 (100.00%)	---	---	---	---	---	---	---	---
Living	alone	10 (52.63%)	1	ref.			1	ref.		
	With a spouse or partner	27 (56.25%)	1.157	0.399	3.36	0.788	4.239	0.631	28.467	0.137
	With family	30 (90.91%)	9	2.029	39.918	0.004	21.485	2.25	205.16	0.008
Salary	PLN 2101 or more	38 (76.00%)	1	ref.			1	ref.		
	PLN 1801–2100	15 (50.00%)	0.316	0.12	0.83	0.019	0.368	0.116	1.161	0.088
	PLN 1501–1800	5 (45.45%)	0.263	0.068	1.018	0.053	0.739	0.086	6.334	0.783
	PLN 1201–1500	5 (100.00%)	---	---	---	---	---	---	---	---
	PLN 901–1200	4 (100.00%)	---	---	---	---	---	---	---	---
Comorbidities	2 diseases	11 (55.00%)	1	ref.			1	ref.		
	3 diseases	39 (66.10%)	1.595	0.568	4.481	0.375	1.275	0.366	4.437	0.703
	4 or more diseases	17 (80.95%)	3.477	0.857	14.113	0.081	2.991	0.547	16.347	0.206

PLN—Polish Zloty; 1 PLN = 0.22 EUR.

**Table 7:** Results of univariable and multivariable regression analysis—multimorbidity and ADL.

Characteristic		N (%)	One-Factor Model				Multivariable Model			
			OR	95% CI		*p*-Value	OR	95% CI		*p*-Value
Sex	Women	17 (32.69%)	1	ref.			1	ref.		
	Men	12 (25.00%)	0.686	0.287	1.643	0.398	0.731	0.272	1.964	0.534
Age	Up to 70 years old	15 (30.61%)	1	ref.			1	ref.		
	71–75 years old	6 (20.00%)	0.567	0.192	1.671	0.303	0.486	0.148	1.593	0.233
	Over 75 years old	8 (38.10%)	1.395	0.478	4.066	0.542	1.451	0.351	6	0.608
Living	Alone	2 (10.53%)	1	ref.			1	ref.		
	With a spouse or a partner	12 (25.00%)	2.833	0.57	14.092	0.203	4.437	0.509	38.676	0.177
	With family	15 (45.45%)	7.083	1.405	35.7	0.018	7.397	0.75	72.93	0.087
Salary	PLN 2101 or more	20 (40.00%)	1	ref.			1	ref.		
	PLN 1801–2100	5 (16.67%)	0.3	0.098	0.914	0.034	0.311	0.093	1.037	0.057
	PLN 1501–1800	3 (27.27%)	0.563	0.133	2.38	0.434	1.627	0.204	12.952	0.645
	PLN 1201–1500	1 (20.00%)	0.375	0.039	3.605	0.396	0.431	0.028	6.754	0.549
	PLN 901–1200	0 (0.00%)	---	---	---	---	---	---	---	---
Comorbidities	2 diseases	4 (20.00%)	1	ref.			1	ref.		
	3 diseases	17 (28.81%)	1.619	0.472	5.55	0.443	1.684	0.435	6.519	0.45
	4 or more diseases	8 (38.10%)	2.462	0.604	10.04	0.209	2.019	0.428	9.532	0.375

PLN—Polish Zloty; 1 PLN = 0.22 EUR.

## Data Availability

The data will be available by contacting the corresponding author.

## References

[1] Ofori-Asenso R., Chin K.L., Curtis A.J., Zomer E., Zoungas S., Liew D. (2019). Recent Patterns of Multimorbidity among Older Adults in High-Income Countries. Popul. Health Manag..

[2] Piotrowicz K. (2013). Opieka ukierunkowana na starszego pacjenta z wielochorobowością—podejście zaproponowane przez Panel Ekspertów Amerykańskiego Towarzystwa Geriatrycznego. Gerontol. Pol..

[3] LaMonica H.M., Hickie I.B., Ip J., Ireland C., Mowszowski L., English A., Glozier N., Naismith S.L. (2019). Disability in older adults across the continuum of cognitive decline: Unique contributions of depression, sleep disturbance, cognitive deficits and medical burden. Int. Psychogeriatr..

[4] Nguyen H., Manolova G., Daskalopoulou C., Vitoratou S., Prince M., Prina A.M. (2019). Prevalence of multimorbidity in community settings: A systematic review and meta-analysis of observational studies. J. Comorbidity.

[5] Williams J.S., Egede L.E. (2016). The Association Between Multimorbidity and Quality of Life, Health Status and Functional Disability. Am. J. Med. Sci..

[6] Yarnall A.J., Sayer A.A., Clegg A. (2017). New horizons in multimorbidity in older adults. Age Ageing.

[7] Fisher K.A., Griffith L.E., Gruneir A., Upshur R., Perez R., Favotto L., Nguyen F., Markle-Reid M., Ploeg J. (2021). Effect of socio-demographic and health factors on the association between multimorbidity and acute care service use: Population-based survey linked to health administrative data. BMC Health Serv. Res..

[8] Wieczorowska-Tobis K., Kostka T., Borowicz A.M. (2011). Fizjoterapia w Geriatrii.

[9] Fidecki W., Wysokiński M., Wrońska I., Kędziora-Kornatowska K., Książkiewicz-Cwyl A., Misiarz J., Kornatowski M. (2018). Wybrane elementy oceny sprawności funkcjonalnej osób starszych. Geriatria.

[10] Manini T. (2011). Development of Physical Disability in Older Adults. Curr. Aging Sci..

[11] Quiñones A.R., Markwardt S., Botoseneanu A. (2016). Multimorbidity Combinations and Disability in Older Adults. J. Gerontol. Ser. A Biol. Sci. Med. Sci..

[12] Stenholm S., Westerlund H., Head J., Hyde M., Kawachi I., Pentti J., Kivimäki M., Vahtera J. (2015). Comorbidity and functional trajectories from midlife to old age: The health and retirement study. J. Gerontol. Ser. A Biol. Sci. Med. Sci..

[13] Pivetta N.R.S., Marincolo J.C.S., Neri A.L., Aprahamian I., Yassuda M.S., Borim F.S.A. (2020). Multimorbidity, frailty and functional disability in octogenarians: A structural equation analysis of relationship. Arch. Gerontol. Geriatr..

[14] Wong C.H., Weiss D., Sourial N., Karunananthan S., Quail J.M., Wolfson C., Bergman H. (2010). Frailty and its association with disability and comorbidity in a community-dwelling sample of seniors in Montreal: A cross-sectional study. Aging Clin. Exp. Res..

[15] Sacha M., Sacha J. (2017). Zespół kruchości—Podejście jedno-i wielowymiarowe. Geriatria.

[16] Uchmanowicz I., Lisiak M., Jankowska-Polańska B. (2014). Narzędzia badawcze stosowane w ocenie zespołu kruchości. Gerontol. Pol..

[17] Uchmanowicz I., Jankowska-Polańska B., Uchmanowicz B., Kowalczuk K., Gobbens R.J.J. (2016). Validity and Reliability of the Polish Version of the Tilburg Frailty Indicator (TFI). J. Frailty Aging.

[18] Huang Y.Q., Gou R., Diao Y.S., Yin Q.H., Fan W.X., Liang Y.P., Chen Y., Wu M., Zang L., Li L. (2014). Charlson comorbidity index helps predict the risk of mortality for patients with type 2 diabetic nephropathy. J. Zhejiang Univ. Sci. B.

[19] Charlson M., Szatrowski T.P., Peterson J., Gold J. (1994). Validation of a combined comorbidity index. J. Clin. Epidemiol..

[20] Charlson M.E., Charlson R.E., Peterson J.C., Marinopoulos S.S., Briggs W.M., Hollenberg J.P. (2008). The Charlson comorbidity index is adapted to predict costs of chronic disease in primary care patients. J. Clin. Epidemiol..

[21] Wysokiński M., Fidecki W., Gębala S. (2013). Ocena samodzielności osób starszych hospitalizowanych na oddziałach internistycznych. Gerontol. Pol..

[22] Michałkiewicz H., Wróbel J. (2012). Zamojskie Studia i Materiały Całościowa ocena geriatryczna—Narzędzie ważne również dla fizjoterapeutów. Zamość Zamoj. Studia I Mater..

[23] RCT (2021). R: A Language and Environment for Statistical Computing.

[24] Wieczorowska-Tobis K., Talarska D. (2017). Geriatria i Pielęgniarstwo Geriatryczne.

[25] Rivera-Almaraz A., Manrique-Espinoza B., Ávila-Funes J.A., Chatterji S., Naidoo N., Kowal P., Salinas-Rodríguez A. (2018). Disability, quality of life and all-cause mortality in older Mexican adults: Association with multimorbidity and frailty. BMC Geriatr..

[26] Fried L.P., Tangen C.M., Walston J., Newman A.B., Hirsch C., Gottdiener J., Seeman T., Tracy R., Kop W.J., Burke G. (2001). Frailty in older adults: Evidence for a phenotype. J. Gerontol. Ser. A Biol. Sci. Med. Sci..

[27] Papachristou E., Wannamethee S.G., Lennon L.T., Papacosta O., Whincup P.H., Iliffe S., Ramsay S.E. (2017). Ability of Self-Reported Frailty Components to Predict Incident Disability, Falls, and All-Cause Mortality: Results From a Population-Based Study of Older British Men. J. Am. Med. Dir. Assoc..

[28] Avila-Funes J.A., Medina-Campos R.H., Tamez-Rivera O., Navarrete-Reyes A.P., Amieva H. (2014). Frailty Is Associated with Disability and Recent Hospitalization in Community-Dwelling Elderly: The Coyoacan Cohort. J. Frailty Aging.

[29] St John P.D., Tyas S.L., Menec V., Tate R., Griffith L. (2019). Multimorbidity predicts functional decline in community-dwelling older adults. Can. Fam. Physician.

[30] Boeckxstaens P., Vaes B., Legrand D., Dalleur O., De Sutter A., Degryse J.M. (2015). The relationship of multimorbidity with disability and frailty in the oldest patients: A cross-sectional analysis of three measures of multimorbidity in the BELFRAIL cohort. Eur. J. Gen. Pract..

[31] Wang Z., Peng W., Li M., Li X., Yang T., Li C., Yan H., Jia X., Hu Z., Wang Y. (2021). Association between multimorbidity patterns and disability among older people covered by long-term care insurance in Shanghai, China. BMC Public Health.

[32] Su P., Ding H., Zhang W., Duan G., Yang Y., Chen R., Duan Z., Du L., Xie C., Jin C. (2016). The association of multimorbidity and disability in a community-based sample of elderly aged 80 or older in Shanghai, China. BMC Geriatr..

